# Exploring the spatial dimension of estrogen and progesterone signaling: detection of nuclear labeling in lobular epithelial cells in normal mammary glands adjacent to breast cancer

**DOI:** 10.1186/1746-1596-9-S1-S11

**Published:** 2014-12-19

**Authors:** Anne Grote, Mahmoud Abbas, Nina Linder, Hans H Kreipe, Johan Lundin, Friedrich Feuerhake

**Affiliations:** 1 Institute for Molecular Medicine Finland (FIMM), Tukholmankatu 8, 00290, Helsinki, Finland; 2 Hannover Medical School (MHH), Institute for Pathology, Neuropathology, Carl-Neuberg-Str. 1, 30625 Hannover, Germany; 3 Albert-Ludwigs-University Freiburg, University Clinic, Institute for Neuropathologie, Breisacher Str. 64, 79106 Freiburg, Germany

## Abstract

**Background:**

Comprehensive spatial assessment of hormone receptor immunohistochemistry staining in digital whole slide images of breast cancer requires accurate detection of positive nuclei within biologically relevant regions of interest. Herein, we propose a combination of automated region labeling at low resolution and subsequent detailed tissue evaluation of subcellular structures in lobular structures adjacent to breast cancer, as a proof of concept for the approach to analyze estrogen and progesterone receptor expression in the spatial context of surrounding tissue.

**Methods:**

Routinely processed paraffin sections of hormone receptor-negative ductal invasive breast cancer were stained for estrogen and progesterone receptor by immunohistochemistry. Digital whole slides were analyzed using commercially available image analysis software for advanced object-based analysis, applying textural, relational, and geometrical features. Mammary gland lobules were targeted as regions of interest for analysis at subcellular level in relation to their distance from coherent tumor as neighboring relevant tissue compartment. Lobule detection quality was evaluated visually by a pathologist.

**Results:**

After rule set optimization in an estrogen receptor-stained training set, independent test sets (progesterone and estrogen receptor) showed acceptable detection quality in 33% of cases. Presence of disrupted lobular structures, either by brisk inflammatory infiltrate, or diffuse tumor infiltration, was common in cases with lower detection accuracy. Hormone receptor detection tended towards higher percentage of positively stained nuclei in lobules distant from the tumor border as compared to areas adjacent to the tumor. After adaptations of image analysis, corresponding evaluations were also feasible in hormone receptor positive breast cancer, with some limitations of automated separation of mammary epithelial cells from hormone receptor-positive tumor cells.

**Conclusions:**

As a proof of concept for object-oriented detection of steroid hormone receptors in their spatial context, we show that lobular structures can be classified based on texture-based image features, unless brisk inflammatory infiltration disrupts the normal morphological structure of the tubular gland epithelium. We consider this approach as prototypic for detection and spatial analysis of nuclear markers in defined regions of interest. We conclude that advanced image analysis at this level of complexity requires adaptation to the individual tumor phenotypes and morphological characteristics of the tumor environment.

## Background

The immunohistochemical detection of estrogen receptor (ER) and progesterone receptor (PR) is an indispensable part of the routine workup of breast cancer in diagnostic pathology providing critical prognostic information, and guiding clinical decisions [1]. The conventional approach of ER/PR scoring in brightfield microscopy by "H-score", "Allred Score", or "Quick Score" [2,3], or according to recommendations of an international expert panel [4] can be complemented by semi-automated computer-aided analysis on whole slide digital images (WSI), either by open source tools [5], or commercially available image analysis software packages [6,7]. These approaches have in common that experienced breast pathologists have to manually annotate regions of interest, either to define the complete area for evaluation, or to train machine-learning algorithms that subsequently recognize corresponding tissue patterns in a "black box"-like way, based on features not known to the user.

While commercially available approaches are helpful for an overall assessment of nuclear staining intensity in individual samples, they do not capture neighborhood relationships of image objects and thus are limited with regard to advanced spatial evaluation of heterogeneous ER and PR expression. In contrast to biomarkers that are constitutively overexpressed due to genomic aberrations, such as HER2/neu, ER and PR expression reflects residual differentiation of tumor cells still sharing commonalities with a potential corresponding "cell of origin" in the normal breast epithelium [8]. Heterogeneous patterns of ER/PR expression in tumors may thus be due to residual transcriptional regulation in response to signals from the tumor microenvironment [9], the menstrual cycle of the patient [10] or after treatment [6]. Image analysis algorithms that enable comprehensive spatial assessment of nuclear markers in their tissue context would support a better understanding of heterogeneous ER/PR expression, with important implications for diagnostic pathology, e.g. for informed decisions on how accurate needle biopsies can represent the hormone receptor status. To analyze such patterns of heterogeneous ER/PR expression in their spatial context, e.g. in relation to adjacent tissue structures, an object-oriented image analysis approach enabling the evaluation of neighborhood relationships and distances between individual image objects is needed.

Herein, we analyze such spatial aspects, capturing ER and PR nuclear positivity, as a proof of concept, in lobules of the mammary gland adjacent to hormone receptor negative breast cancer. We show that object-oriented evaluation of ER/PR expression in the spatial context of surrounding tissue is feasible and propose an approach of automated detection of lobular structures based on texture features, limiting human interaction largely to subsequent quality control. We also discuss potential limitations of this approach, specifically concerning robust automated distinction between pre-existing breast epithelium and well differentiated ER/PR positive breast cancer, and outline perspectives how to overcome such challenges in advanced image analysis by approaches tailored to tumor morphology and immunophenotype. The work supports the development of improved techniques to assess and understand heterogeneity and spatial interactions of ER/PR positive neoplastic and normal tissues.

## Methods

### Material

A training set (n = 6) and a test set (n = 12) of hormone receptor negative ductal invasive breast cancer tissue samples were selected and 2-4 µm thick paraffin sections were cut and stained using automated ER and PR detection (Ventana Benchmark Ultra) with diaminobenzidine (DAB) as chromogen. Slide scanning was performed using a whole-slide scanner at 20× magnification (Aperio ScanScope, Leica Microsystems, Wetzlar, Germany), and saved in a proprietary (svs) format using JPEG2000 compression. In preparation of knowledge-based specific feature selection, image annotation for the purpose of interdisciplinary discussion of relevant tissue structures was performed using the freely available Aperio ImageScope software (Leica Microsystems). Studies were performed on anonymized archival paraffin-embedded material. The study was approved by the local Ethics Committee.

### Method

The image analysis software Developer XD2 (Definiens, Munich, Germany) was used for image analysis. A rule set was developed based on knowledge-based selection of modular image analysis algorithms and parameters, e.g. for segmentation and hierarchical classification, using the functionality of the software package that facilitates translation of recurrent image features assigned to biologically relevant structures into an executable, modular analysis workflow. The whole slide images were first down sampled to 10% from the original resolution of 0.56 microns/pixel, and classified into tissue and background. The next steps were performed only in the tissue regions. First the images were segmented on different coarseness levels; then lobules and tumor were detected. Inside the lobules, the percentage of positive nuclei (ER or PR) was determined for each lobule. The lobules were sorted into three categories according to their distance to tumor tissue and the distribution of positivity was evaluated.

#### Pre-processing

The whole slide images have a pixel size of 0.56 microns/pixel at original scale. As processing of the whole images in original resolution requires inacceptable computational power and processing times, and large scale tissue structures such as tumor mass and lobules appear more salient at lower resolutions, the images were down sampled to 10% of the original resolution, using nearest neighbor resampling, to a working pixel size of 5.6 microns/pixel.

In order to describe textural properties of the image, an additional image layer was calculated. The textural feature used for this is the standard deviation of each pixel to the neighbor pixels. This textural layer was derived from the blue channel. The textural layer was median filtered with a 3 × 3 window and then smoothed with an 11 × 11 Gaussian kernel.

For separation of tissue and background the image was smoothed with a 15 × 15 Gaussian kernel. A threshold was automatically computed on the smoothed image, and pixels higher than the threshold were classified as background. Small areas of background surrounded by tissue were reclassified as tissue and vice versa. All subsequent operations were only performed in the tissue regions.

#### Segmentation

**Figure 1:**
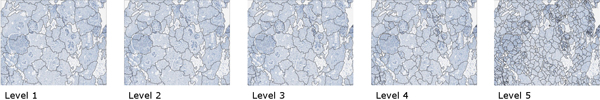
**Hierarchy of segmentation levels**.

In preparation for the classification, the images were segmented at five different levels of granularity, using the multiresolution segmentation described in [11] as implemented in the image analysis software. Multiresolution segmentation aims at creating spectrally homogeneous segments. The level of heterogeneity allowed is controlled by a scale parameter. The higher the value of the scale parameter, the more heterogeneous - and thus larger - the segments are allowed to be. Here, the segmentation started with a rather large scale parameter to produce the first and coarsest level, then the scale parameter was decreased for each subsequent step of the segmentation. The scale parameter was set to 100 for the first segmentation, and decreased in steps of 20 for the following segmentations. The resulting segmentation is hierarchical, such that a segment in a finer level can be a sub-segment of only one segment in a coarser level (Figure 1). The five different levels were created as preparation for the lobule classification, where the combined segment properties of different levels are used. As spectral information to compute the homogeneity criterion, the RGB image channels were used in combination with the computed texture channel. The texture channel was weighted twice as much as the color channels, thereby placing emphasis on the textural features that separate lobules from non-lobules.

#### Initial lobule classification

The classification of lobules was performed in two steps. In the first step, the initial lobule classification, lobule candidates were classified based on textural, geometric and relational features in the four coarsest segmentation levels independently. Rationales for feature selection were two-fold: (1) based on visual assessment of previously annotated lobular structures in different image channels, and (2) biological knowledge such as tubular structures, reflected by regular intensity variation in images.

**Figure 2:**
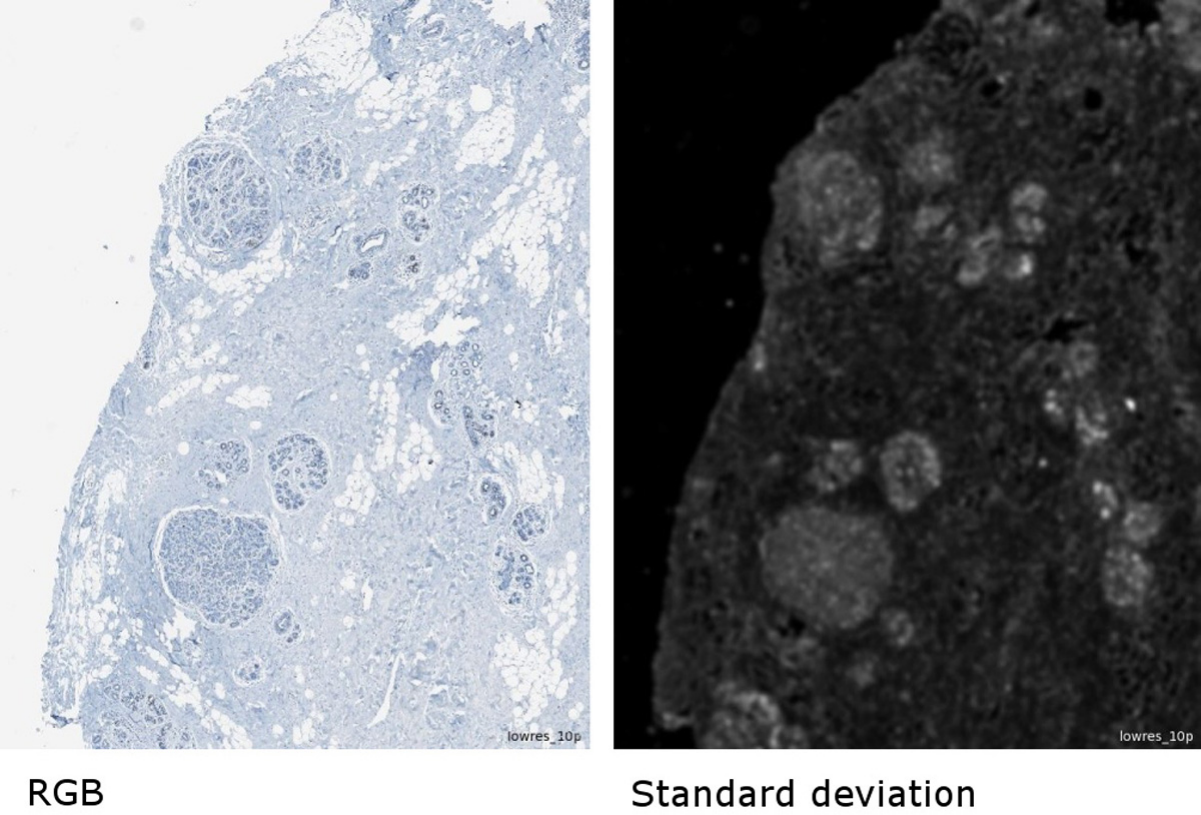
**Original RGB image subset and standard deviation to neighbors texture channel**.

As *textural feature*, the mean (per segment) of the standard deviation to neighbor pixels (in the following abbreviated as stddev-n) was used, which describes the local variance of intensity values. While stromal tissue usually shows a rather low stddev-n, normal lobules could be observed to have a higher stddev-n within a fairly constant range of 15-36. At the low resolution, where nuclear diameter corresponded roughly to one-two pixels, this probably reflected the lobules' regular internal structure consisting of rings of epithelial cells. This property caused the lobules to stand out in the stddev-n channel as relatively bright blobs against a darker stromal background (Figure 2).

As *geometric features*, the area and roundness of the segments were used, reflecting the fact that most sections of lobules were within a certain area size (range, 2000-14000 square pixels, i.e. 0.0627-0.439 mm^2^), and their shape appeared relatively round, or oval. Roundness was defined here as the difference between the radii of the largest enclosed ellipse and the smallest enclosing ellipse, where a smaller value corresponds to more roundness, 0 meaning a perfect ellipse (accepted range, 0-1.3).

The *relational features *describe the relations of a segment to its neighbor segments. Here spectral relations derived from the textural layer are examined. The difference of means in the stddev-n channel to neighbor segments and the border contrast to neighbor segments are used as relational features, reflecting the fact that the lobules are brighter than their surroundings in the stddev-n channel. Consequently, both the difference of means and the border contrast have to exceed a minimum (difference of means >= 5; border contrast >= 0.4).

All conditions for the features must be met for a segment to be classified as lobule candidate. After the initial classification, lobule candidates have been identified in the four coarsest segmentation levels.

#### Refined lobule classification

The initial classification, where segments were classified as lobules in each segmentation level independently, still contained many errors: segments falsely classified as lobules, missed lobules, lobule regions merged with surrounding regions, missing parts of lobules. Therefore, the initial lobule classification was refined based on the properties of sub-segments, starting with the coarsest segmentation level and working through the finer levels. Each lobule candidate was evaluated using the following rules: if the sub-segments were too different from each other, the lobule classification was removed. If all sub-segments fulfilled a subset of criteria for lobule candidates (without the conditions on minimum area and border contrast), the lobule was accepted as a whole. If a sub-segment did not fulfill the criteria, and at least half of its border corresponded to the border of the lobule (thus being more at the outside), it was removed from the lobule. The remaining segments were merged, and if the merged segment fulfilled the full set of criteria for lobules (as in the initial classification) it was accepted as a lobule. In this way, the lobule classifications were consolidated and carried through the levels to the fourth level. After classification refinement, this level contained the final lobule classification and was used in the subsequent steps. As a last step, adjacent segments which fulfilled the subset of lobule criteria were merged and reclassified as lobule if the merged segment fulfilled all lobule criteria. For evaluation of the detection quality, a pathologist manually labeled detected lobules as true positive or false positive detections, and missed lobules as false negatives, re-classifying incorrectly detected structures within the user interface of the software package.

#### Tumor classification

Choosing cases with hormone receptor negative tumor for initial proof of feasibility, tumor areas were classified based on adding segments to seed segments, assuming a tumor with coherent tumor mass. In order to find seed segments in the down sampled images, the median of the mean stddev-n values was determined from all tissue segments which were not classified as lobules. Segments whose mean stddev-n values were very close to the median (± 0.1) were selected as seed segments, as in our samples these had a high probability to be located inside the tumor. After detecting the seed segments, neighbor segments were added iteratively on two conditions: their mean stddev-n should be close to that of the already detected tumor region (difference of at most 2) and the shared border to the tumor region should be at least 20% of the total segment border. In a post-processing step, holes inside the tumor region were closed and isolated tumor regions smaller than 1/3 of the largest tumor area eliminated, based on the fact that these usually were false detections, especially occurring at the tissue borders where the tissue was folded.

#### Nuclei detection in lobules

In order to detect positive (brown DAB signal) and negative (blue hematoxylin signal) nuclei and calculate the percentage of positive nuclei inside the lobules, each lobule was processed separately on the original resolution of 0.56 µm/pixel. First, brown DAB positive and blue DAB negative stain channels were separated using an implementation of a color deconvolution algorithm which uses a hue-saturation-density model for stain recognition [12]. Nuclei were detected in both channels separately. For the nuclei detection, preliminary nuclear regions were determined pixel-based where the intensity value in the respective stain channel is higher than an empirically determined threshold (0.2) as well as higher than that of the other stain channel. A watershed algorithm within the nuclear regions, applied to the respective stain channels, was then used to separate adjacent nuclei. The separated regions formed by the watershed algorithm needed to have a minimum area to be accepted as nuclei. Holes in detected nuclear objects were filled. Since some nuclei can remain merged in the watershed segmentation due to insufficient spectral border differences, in the next step merged nuclei were separated based on geometry: they were cut at significant dents. Significant dents are dents where the angle between the two tangents starting at the corner pixel is more than 30 degrees. In post-processing, the borders of nuclei were smoothed, and detected nuclei whose area was smaller than the minimum nuclear area were removed.

#### Evaluation of nuclear positivity in lobules

The nuclear positivity for ER and PR in the lobular epithelium was evaluated in relation to their distance to the tumor. For this purpose, Lobules were divided into three groups based on their distance to the tumor: adjacent (<0.5 mm), intermediate (0.5-2 mm) and distant (>2 mm). Distances were measured from the center of the lobule to the nearest tumor border.

The specific functionalities of the Image Miner® software (Definiens) were used to display the results in table format, to visualize potential trends observed for ER/PR positivity per lobule in relation to the lobules' distance from the tumor in graphics, and for linking individual regions of interest (in this case, individually analyzed lobules) with corresponding data points in tables or graphical displays. The latter functionality was used to facilitate a fast review of individually analyzed regions of interest in the context of comprehensively visualized data.

## Results

### Feasibility and accuracy of lobule detection

**Figure 3:**
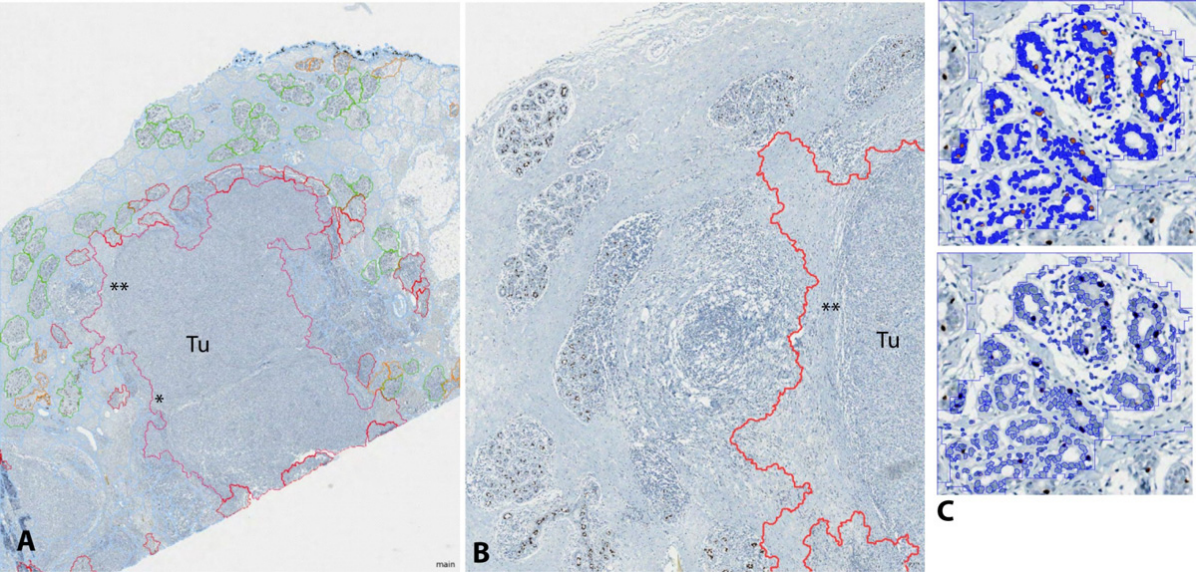
**Accuracy of lobule detection and PR nuclear positivity in a test case**. **A **Accuracy of lobule detection is best at intermediate distance to the tumor (Tu). Note the false positive structures (circled in red, manually excluded from analysis), initially misclassified as lobules within a zone of inflammatory tumor microenvironment. **B **Detail showing the effect of inaccuracies in tumor classification. Most of the tumor borders are correctly detected (*), but in a small stretch the algorithm has classified too much of the surrounding stroma as tumor area (**). As a consequence, the distance to tumor borders may be slightly underestimated in such regions. **C **Upsampling of lobule areas as individual regions of interest within the workflow reduces the areas subjected to subcellular analysis of nuclei at high resolution.

We identified the mean standard deviation to neighbor pixels within a segment as a critical textural feature for lobule detection. This relatively simple feature was found to be a strong discriminator between lobules and the surrounding tissue, as visually assessed by comparison of the lobule outlines in the newly generated texture channel as compared to the corresponding RGB channels of the original images. Its values for lobules appear to be relatively stable within a certain range, which most likely reflects the regular morphology of tubular glandular structures with luminal compartments alternating with more compact basal layers. As additional geometric features, we found useful the size of lobule candidates and a minimum roundness criterion. The relational features were coupled to the textural features: border contrast and mean difference, both calculated for the mean standard deviation, were used to evaluate the relations to neighbor segments. After rule set optimization in the training set, the test set showed acceptable automated lobule detection quality (true positive regions of interest ranging from 40-80%, false negative lobules outlined accurately enough in the segmentation for fast re-classification) in 4/12 (33%) of new cases. The commonality of the 8 cases with lower detection quality was widespread brisk inflammatory infiltrate in the tumor periphery, disrupting the lobular structures (so-called "lymphocytic lobulitis") (Figure 3A).

### ER/PR expression in lobular epithelium in relation to distance from tumor

**Figure 4:**
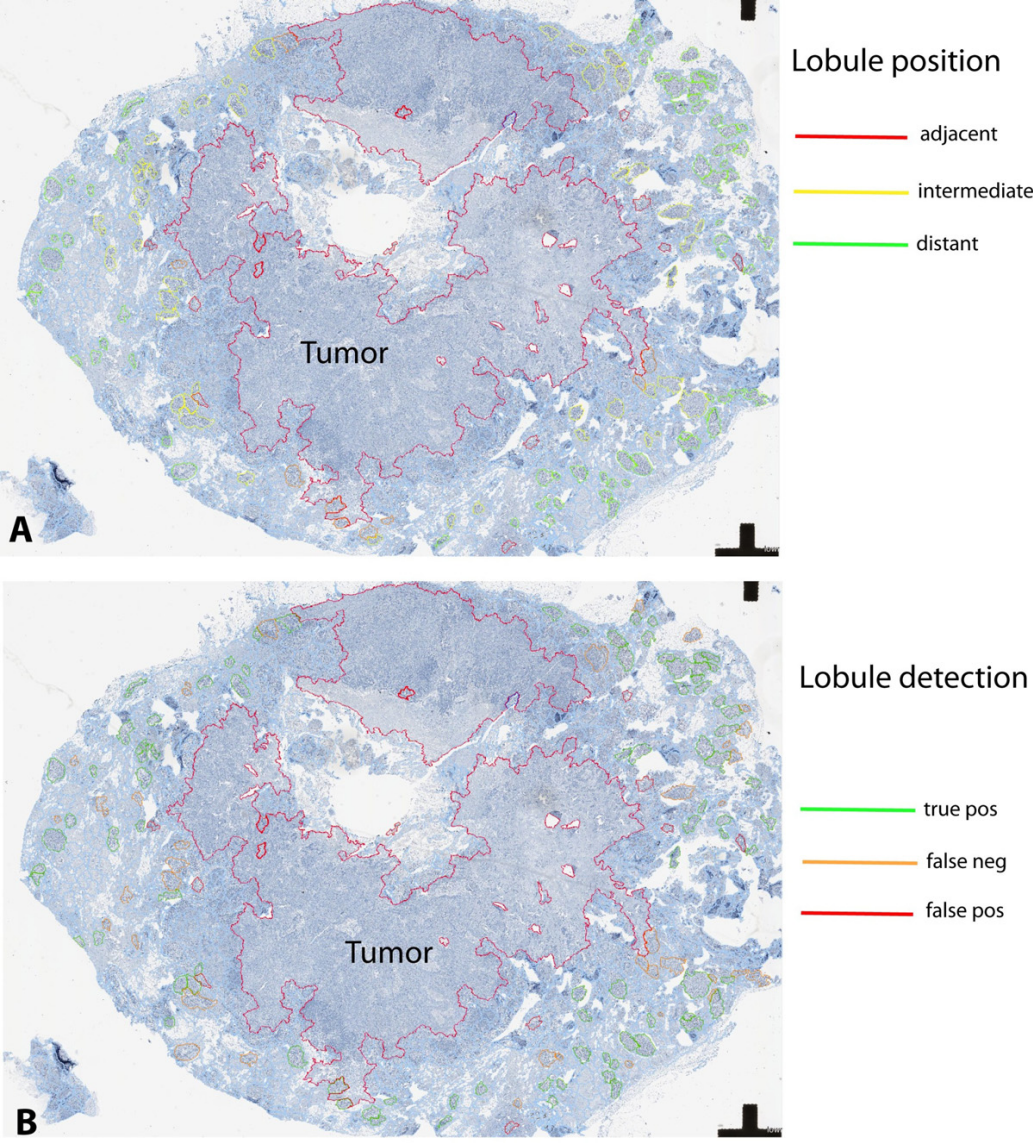
**Spatial context of ER/PR signaling**. Graphical display of normal mammary lobules grouped into categories according to distance to tumor (A) and detection accuracy (B) as defined by a pathologist's annotation of lobules in a training case.

In contrast to irregular tumor growth, seemingly forming random substructures, pre-existing breast tissue has a well-defined structure and distribution of hormone-receptor positive cells. Thus, the rationale for using predefined features in ER/PR evaluation in breast lobules was their clearly defined shape within a relatively consistent 3-dimensional structure and a regular distribution throughout tumor adjacent tissue. For automated evaluation of ER/PR in the spatial tissue context, we selected for a first proof-of-concept those study samples that contained coherent tumor masses with clearly defined invasive edges. We started to develop automated tumor detection for ER/PR negative breast cancer, in order to start with a morphological and immunohistochemical phenotype sufficiently distinctive from normal mammary gland epithelium. In images with this kind of tumor, the detection quality was sufficient for the purpose of estimating lobule distances from the tumor border in images, although the tumor border was not always correctly delineated (Figure 3B). The lobules were manually labeled as "correctly detected" (true positive, TP), "falsely detected" (false positive, FP) or "missed" (false negative, FN; see Figure 4B). In order to evaluate the ER/PR expression independently from the quality of the lobule detection, only lobules labeled as correctly detected or missed (and retrospectively labeled as lobules) were included in this evaluation. These lobules were sorted into the three categories "adjacent", "intermediate" and "distant" according to their distance to the tumor border (Figure 4A).

A total of 551 lobules (ER) and 530 lobules (PR) were evaluated. Overall, 51 (ER) and 42 (PR) lobules were adjacent to the tumor (<0.5 mm), 187 (ER) and 214 (PR) lobules were at an intermediate distance 0.5-2 mm, and 313 (ER) and 274 (PR) lobules were distant (>2 mm). We observed that overall the ER and PR nuclear staining tended to be lower in close proximity to the tumor bulk as compared to distant lobular structures (Tables 1 &2). Taking into consideration the preliminary nature of our approach as prototypic example for automated selection of region-specific image analysis, we did not seek formal statistical confirmation of a potential gradient of ER/PR labeling. Also, we observed some deviations from the general trend, for example in case 2 that included fewer lobules in total. Case 4 was different from the rest, because it was largely negative for PR in lobular structures, while some positive nuclei were seen in the ER staining, indicating that ER and PR regulation may be detached from each other. An additional rationale not to perform formal statistical analysis on this pilot data set was the fact that the ER images from case 5 and 6 were used for method development.

**Table 1:** Summary of average ER positivity in relation to the tumor neighborhood.

ER	Average ER^+ ^nuclei in adjacent lobules	number adjacent lobules	Average ER^+ ^nuclei in intermediate lobules	number intermediate lobules	Average ER^+ ^nuclei in distant lobules	number distant lobules
Case 1	6.5%	7	14.4%	31	20.2%	21

Case 2	11.0%	8	16.9%	15	13.7%	10

Case 3	-	0	26.1%	47	28.3%	94

Case 4	9.6%	24	12.9%	20	14.9%	25

Case 5 (train)	2.8%	9	4.8%	49	7.3%	68

Case 6 (train)	4.5%	3	6.6%	25	17.0%	95

**Table 2:** Summary of average PR positivity in relation to the tumor neighborhood.

PR	Average PR^+ ^nuclei in adjacent lobules	number adjacent lobules	Average PR^+ ^nuclei in intermediate lobules	number intermediate lobules	Average PR^+ ^nuclei in distant lobules	number distant lobules
Case 1	5.7%	7	10.6%	35	10.6%	18

Case 2	2.5%	3	11.2%	12	7.4%	4

Case 3	11.8%	12	9.2%	70	10.4%	74

Case 4	0.4%	1	0.3%	12	0.7%	73

Case 5	4.2%	6	8.2%	43	10.7%	49

Case 6	4.5%	13	7.2%	42	7.4%	56

In order to develop methods robust enough for applications in translational clinical studies, we used real clinical material instead of specifically processed consecutive slides. As a consequence, several sectioning levels could be between the ER and PR stained slides, explaining different numbers of lobules, or lobular segments across slides. Besides the impact of sectioning levels, variable thickness and variation in staining/counterstaining intensity, and processing artifacts could contribute to variations in the detection quality.

### "Image mining" approach to quality control and comprehensive assessment of ER/PR spatial distribution

**Figure 5:**
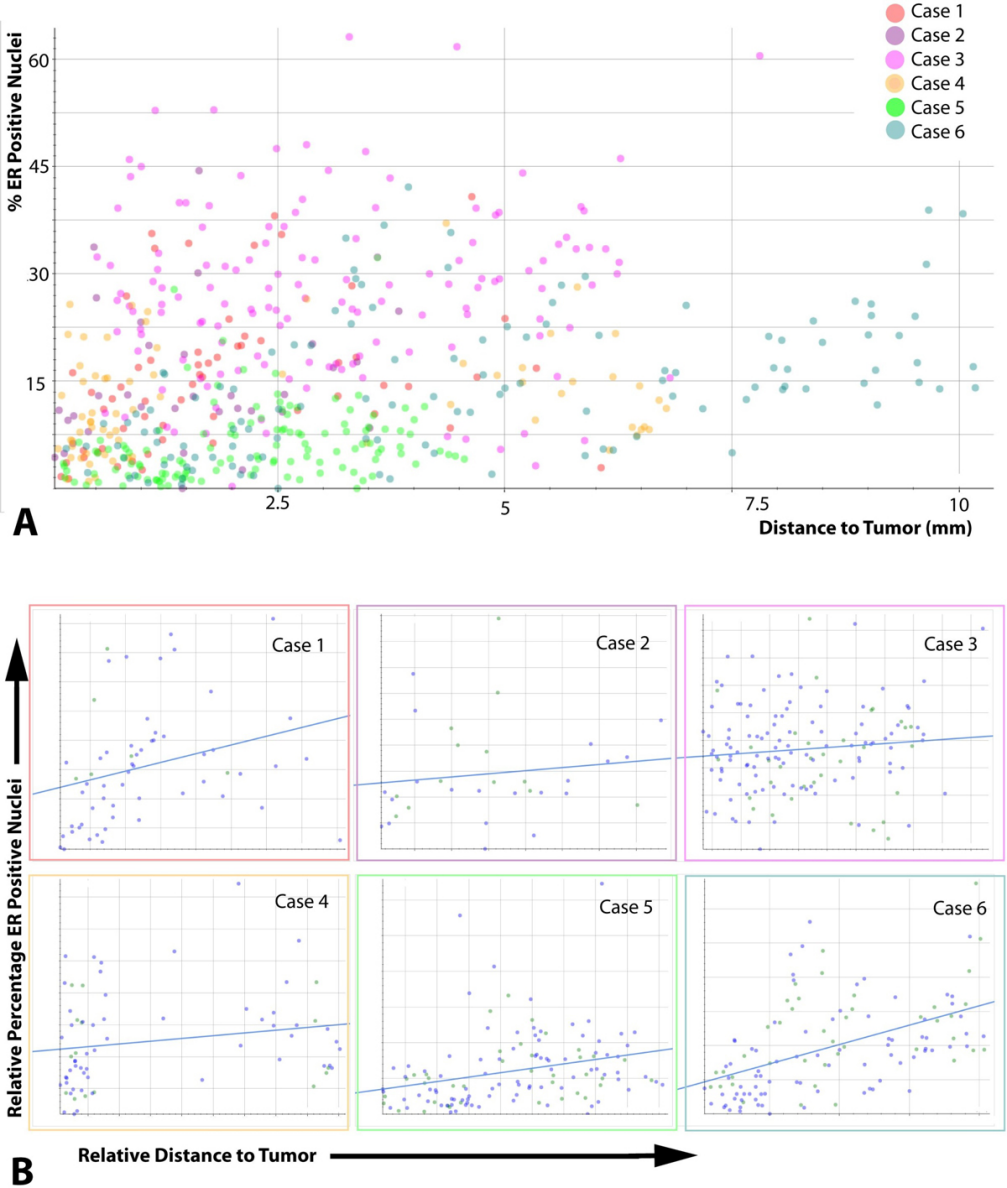
**Connecting data points with regions of interest for individual assessment of lobules**. Image analysis approaches connecting data points with individual automatically detected regions of interest enable individual assessment of lobules, pattern detection and quality control. **A **Overview on analysis results of six cases stained for ER. Every dot reflects the ER positivity ratio in an individual lobule. The spatial distribution of lobules, their predominant staining behavior and the occurrence of outliers is visible at a glance and can be connected with the corresponding upsampled images. **B **Per-case view. Each dot represents one lobule in the respective case. The horizontal axis shows distance to tumor, the vertical axis shows positive percentage. The blue lines are regression lines.

**Figure 6:**
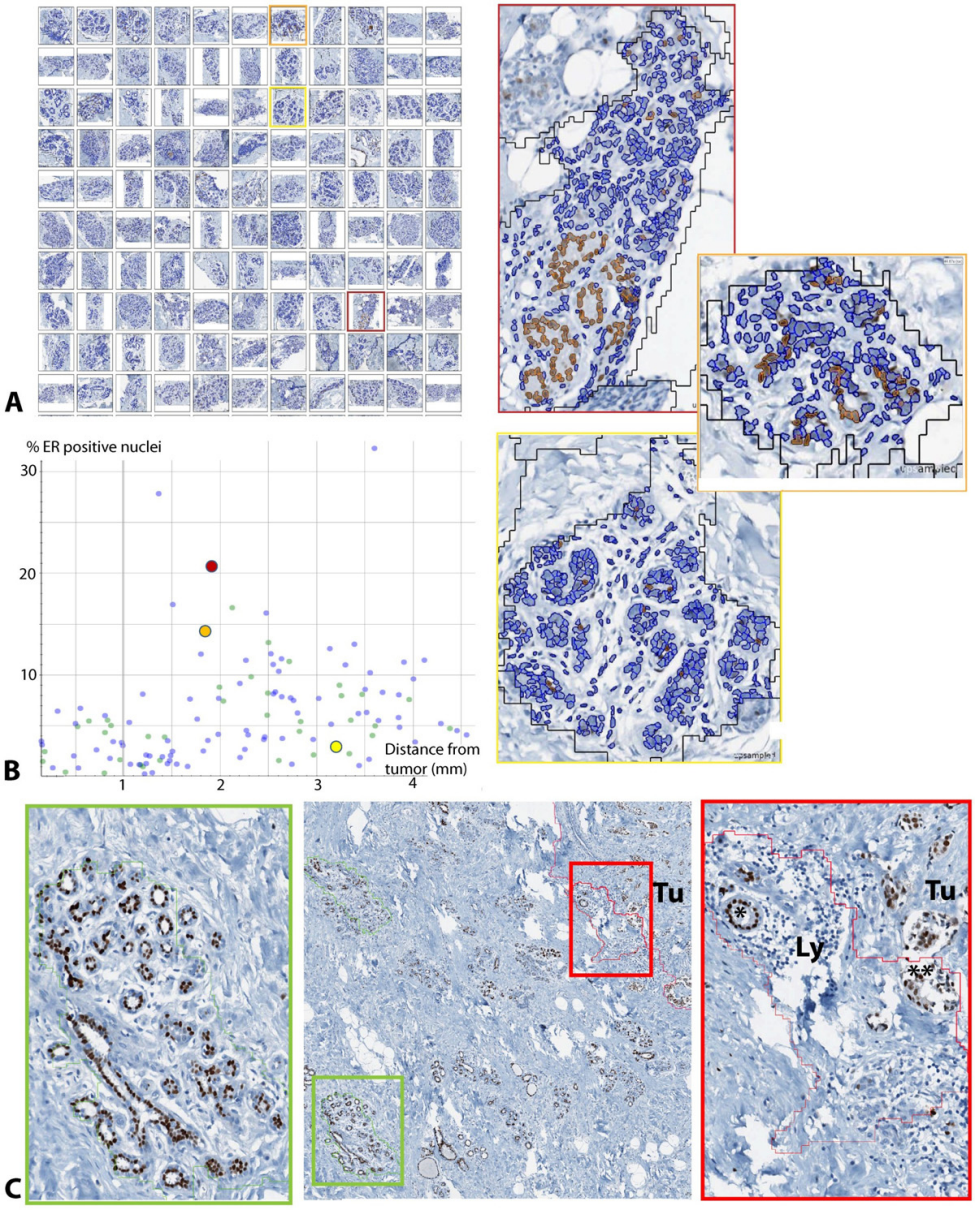
**Quality control by linking data points with regions of interest**. Directly linking data points with regions of interest facilitated the evaluation of outliers, exclusion of technical errors, and quality control of the nuclei detection at high resolution. Snapshots of all analyzed lobules in one case labeled in **A **("Image gallery") with a colored frame, were tracked in the data plot **B**, and enlarged for quality control. Note that each individual positive nucleus can be investigated as single image object in their spatial context, for example with regard to clusters of positive cells (orange frame) as compared to diffuse distribution (yellow frame). **C **Example for an ER positive case with an accurately detected lobule (green frame) and a false positive (red frame), the latter revealing some conceptual challenges in this tumor subtype, such as the distinction between pre-existing, reactively altered tubular structures (*) and ER positive tumor cells with glandular differentiation (**)

Analyzing each lobular structure as individual region of interest within a WSI (Figure 3C) enabled a comprehensive overview on the data structure, helped identifying outliers, and facilitated the recognition of recurrent staining patterns. For this purpose, we used a graphical display of all analyzed lobules in relation to the distance to the tumor, confirming a recurrent pattern of decreasing ER/PR expression in immediate neighborhood to the tumor (Figure 5). For detailed analysis of outliers, a quality control (QC) image of each analyzed lobule was exported, and linked with corresponding data points in the graphical display. This approach confirmed robustness of both lobule and nuclei detection, and allowed a visual assessment of potential errors, e.g. the admixture of infiltrating immune cells, or the inclusion of ER/PR positive ductal structures (Figure 6A&B). In a small pilot set for the analysis of hormone receptor positive breast cancer, our approach revealed specific challenges, due to the similarity between well differentiated breast tumor formations and morphologically altered glandular structures in the immediate neighborhood of the tumor invasive edge (Figure 6C).

### Computation times

The computation time for the preprocessing, segmentation and classification steps depends on the size of the input image, while the time for the detailed lobule analysis depends on the number of lobules and the number of nuclei that are detected inside the lobules. On a PC with a 64 bit Windows 7 operating system, with four Intel ® Core™ i5-2400 3.10 GHz processors and 16 GB RAM, the computation up to the classification step took between 4 and 25 minutes for our images, which were in a size range between 400 and 1800 Megapixels. The lobule evaluation took on average 30 seconds per lobule, for an average number of 1800 nuclei per lobule and an average lobule size of 0.24 mm^2^. The number of processed lobules per image varied from 19 to 156, resulting in processing times for the lobule evaluation between 10 minutes and 1 hour 20 minutes. Total processing times were between 17 minutes and 1 hour 50 minutes.

## Discussion

Accurate detection of ER/PR positive nuclei in the spatial context of surrounding tissue structures is a prerequisite for a comprehensive assessment of hormone receptor signaling both in breast cancer and normal tissues. Our work aims at broadening the available tools for ER/PR evaluation beyond counting positive nuclei in manually annotated regions. We prove feasibility of partially automated detection of biologically relevant regions such as normal lobular structures, and thereby provide guidance how to address similar image analysis challenges, for example region-specific assessment of heterogeneous ER/PR patterns in human tumors, or the analysis of hormone-receptor positive orthotopic xenografts that can be transplanted into pre-existing rodent mammary ducts and glands. While the current practice of conventional ER/PR scoring in clinical routine is reproducible, and widely established as rationale for treatment with anti-estrogen treatments such as aromatase inhibitors [4,13,14], the underlying causes for heterogeneous staining results, potentially due to regulation of ER/PR in response to the tumor microenvironment, are poorly understood. Improved tools to capture and systematically analyze heterogeneous staining patterns will lead to better understanding of the biology and more accurate interpretation of ER/PR staining after therapy [15] and will improve diagnostic accuracy of testing in small samples, such as tissue microarrays (TMA) cores [16], or needle biopsies [9,17].

As a first proof of concept for detection of ER/PR positive nuclei in morphologically defined regions of interest, we showed that lobular structures can be accurately detected in a subset of tumor samples, using automated image analysis based on the presented textural, relational, and geometrical features. We further showed that ER/PR can be analyzed in the context of spatial parameters, such as distribution of the individual regions of interest. Since each positive cell nucleus is captured as an independent image object in the present workflow, our approach would, in principle, enable an extended analysis at subcellular level (ongoing work, preliminary data not shown). A potential limitation to this approach is that evaluation of neighborhood relationships in individual epithelial cells requires the related structures to reside within the respective region of interest. We selected lobular structures because we found recurrent morphological patterns such as tubular structures helpful to define useful image features, and we decided to start our approach with hormone receptor negative breast cancer that, from an image analysis perspective, shares less commonality with normal epithelium than well differentiated hormone receptor expressing tumors. In principle, our approach can also be extended to ER/PR detection in defined compartments of tumor tissue, e.g. the tumor center in contrast to the invasive edge, or to regions adjacent to stromal structures. The translational relevance of such applications of our analysis workflow is supported by previous studies on pathway activation markers, showing that the vicinity to stroma, inflammation, and position within the tissue sample that might influence time to fixation has significant impact on biomarker analyses [18]. Further development of our approach into automated analysis of tumor cells in spatial context of surrounding tissue would substantially broaden the spectrum of computer-assisted ER/PR assessment, reducing the manual interaction of pathologists who annotate individual regions for analysis [6,19].

An important observation was the challenge to automatically detect lobular structures that are disrupted by severe inflammatory response. While this can be seen as an important limitation of our approach as long as the focus is on the accurate detection of epithelial structures, it may also be an important finding with regard to the interaction between tumor-based biomarker studies and the inflammatory tumor microenvironment (iTME). The difficulty identifying lobules exhibiting lymphocytic lobulitis [20,21] is, from an image analysis perspective, not very different from iTME analyses in well differentiated, predominantly small cell breast cancer subtypes. As the inflammatory tumor microenvironment is an important component of the tumor phenotype in breast cancer [22], and may, as recently shown in colon cancer, even reflect distinctive somatic genomic aberrations in the malignant cells [23], we expect increasing demand for automated analysis methods that include evaluation of the iTME. Therefore, we regard our results as a further step towards comprehensive assessment of both the neoplastic and non-neoplastic tumor compartments. Therefore, ongoing current work aims at integrating ER/PR analysis modules with advanced methods for iTME evaluation [24,25].

Our preliminary approach to check for plausibility of automated cell nucleus detection is currently based on visual assessment of classification results in single lobules. While this is largely sufficient for a pragmatic estimation of the detection of positive cell nuclei at high resolution, we aim at more formal ways to test for accuracy and to define acceptable detection rates of mammary lobules. Nevertheless, our results suggest that the robust detection of the biologically relevant regions of interest may be a greater bottleneck for further automation of WSI - based ER/PR analysis than the analysis of nuclear staining, once the regions of interest are defined. Herein, we propose a stepwise analysis approach at different levels of image resolution, resulting in a reasonable balance between the detection accuracy and processing times. Limitations of this approach can be expected as early in the analysis workflow as at the segmentation step, and current ongoing work addresses this issue by approaches for breast cancer-specific segmentation solutions that are adjusted to the requirements of WSI analysis [26]. Other attempts to tackle the breast cancer specific image analysis requirements include specific solutions for the distinction of ER/PR positive nuclei of well differentiated tubule-forming tumor cells in contrast to physiological ER/PR expression in the epithelial lining of adjacent breast tissue. Complex analysis challenges like this are very specific for breast cancer and likely to require tailored, object-oriented image analysis approaches rather than general tools applicable to different sorts of tumor tissue.

## Conclusions

We have shown that normal lobular structures in whole slide images can be classified at low resolution using textural and geometric features. The object-oriented approach allows both a detailed analysis of each individual lobule and an image-wide analysis of the spatial relationships between lobules and tumor, and enables analysis of the lobules at high resolution, for example for ER/PR positivity, in acceptable processing time, for example enabling overnight batch processing of 10-12 WSI slides at 20× magnification on a normal PC with current processor technology, and correspondingly higher throughput on work stations with higher computational power. We consider this as a proof of concept that WSI data can be used to comprehensively detect regions of interest and submit those regions automatically to subsequent detailed analysis. Among others, this could be used for broader applications of ER/PR detection, for example facilitating the assessment of heterogeneous ER/PR expression the spatial tissue/tumor context. Ongoing work includes more detailed analysis of regions of interest at subcellular level, broadening the scope of the analysis to different types of breast cancer, and performing more rigorous statistical tests on the detection results.

## Competing interests

F. Feuerhake was full-time employee of Roche Pharma Research and Early Development (pRED) until September 30, 2012. The authors declare no further conflict of interest.

## Authors' contributions

AG: Developed and performed image analysis, performed statistical analysis, wrote manuscript. MA: Supervised immunohistochemistry, selected cases, and annotated regions of interest ("ground truth"). NL: Supervised image analysis, critically revised manuscript. HHK: Supervised study concept and design, performed histopathological evaluation, revised manuscript. JL: Supervised image analysis, revised manuscript critically and provided input on statistical considerations. FF: Developed study conception and design, performed data acquisition, annotated regions of interest ("ground truth"), supervised image analysis and rule set development, performed image analysis, wrote and revised manuscript.
